# rt269I Type of Hepatitis B Virus (HBV) Leads to HBV e Antigen Negative Infections and Liver Disease Progression via Mitochondrial Stress Mediated Type I Interferon Production in Chronic Patients With Genotype C Infections

**DOI:** 10.3389/fimmu.2019.01735

**Published:** 2019-07-24

**Authors:** So-Young Lee, Yu-Min Choi, Song-Ji Oh, Soo-Bin Yang, JunHyeok Lee, Won-Hyeok Choe, Yoon-Hoh Kook, Bum-Joon Kim

**Affiliations:** ^1^Department of Biomedical Sciences, Microbiology and Immunology and Liver Research Institute, College of Medicine, Seoul National University, Seoul, South Korea; ^2^Department of Internal Medicine, Konkuk University School of Medicine, Seoul, South Korea

**Keywords:** hepatitis B virus, HBV e antigen (HBeAg) negative infection, genotype C, mitochondrial stress, type I interferons

## Abstract

Hepatitis B virus infection is a serious global health problem and causes life-threatening liver disease. In particular, genotype C shows high prevalence and severe liver disease compared with other genotypes. However, the underlying mechanisms regarding virological traits still remain unclear. This study investigated the clinical factors and capacity to modulate Type I interferon (IFN-I) between two HBV polymerase polymorphisms rt269L and rt269I in genotype C. This report compared clinical factors between rt269L and rt269I in 220 Korean chronic patients with genotype C infections. The prevalence of preC mutations between rt269L and rt269I was compared using this study's cohort and the GenBank database. For *in vitro* and *in vivo* experiments, transient transfection using HBV genome plasmid and HBV virion infection using HepG2-hNTCP-C4 and HepaRG systems and hydrodynamic injection of HBV genome into mice tails were conducted, respectively. This report's clinical data indicated that rt269I vs. rt269L was more significantly related to HBV e antigen (HBeAg) negative serostatus, lower levels of HBV DNA and HBsAg, and disease progression. Our epidemiological study showed HBeAg negative infections of rt269I infections were attributed to a higher frequency of preC mutations at 1896 (G to A). Our *in vitro* and *in vivo* studies also found that rt269I could lead to mitochondrial stress mediated STING dependent IFN-I production, resulting in decreasing HBV replication via the induction of heme-oxygenase-1. In addition, we also found that rt269I could lead to enhanced iNOS mediated NO production in an IFN-I dependent manner. These data demonstrated that rt269I can contribute to HBeAg negative infections and liver disease progression in chronic patients with genotype C infections via mitochondrial stress mediated IFN-I production.

## Introduction

Hepatitis B virus (HBV) infection is a high-risk global health issue leading to severe liver disease. In 2015, it was estimated that 350 million patients were chronically infected worldwide and 900,000 patients died (1). Although vaccines and therapeutic agents are currently available against HBV, the number of deaths caused by HBV has increased worldwide (2).

HBV is an enveloped and partially double-stranded DNA virus and preferentially replicates in hepatocytes. The HBV genome consists of four open reading frames (ORFs): surface antigens (S), core proteins (C), polymerase (Pol), and X proteins (X) (3). After infecting the hepatocytes, the genomes from HBV are converted to covalently closed circular DNA (cccDNA) by host enzymes (4). Since HBV cccDNA cannot be completely eradicated by nucleot(s)ide analog agents (5), epigenetic regulation via innate immune response modulating agents such as Type I IFN (IFN-I) is necessary for the complete viral clearance from chronic hepatitis B (CHB) patients (6).

IFN-I is a first-line defense mechanism of innate immune systems for viral infections (7) that is mediated via host recognition of viral pathogen-associated molecular patterns (PAMPs) through pattern recognition receptors (PRRs) (8). However, for its survival, HBV has developed various strategies to evade host IFN-I dependent innate immunity (9). It has been reported that some HBV proteins such as HBV surface antigen (HBsAg), HBV e antigen (HBeAg), and HBV virions can lead to inhibit Toll-like receptor (TLR) mediated IFN-I production (10). In addition, HBxAg and Pol can also negatively regulate retinoic acid inducible gene I (RIG-I) mediated antiviral responses (11, 12). In RNA sensing pathways, it has also been reported that HBV Pol can block IFN-I dependent antiviral pathways via the inhibition of STING dependent cytosolic DNA sensing pathways (13).

On the basis of an 8% divergence in HBV genome sequences, HBV has been characterized into 10 genotypes as A-J (14). Various studies on HBV genotypes have reported that they have distinct pathogenic potentials as well as distinct geographic and ethnic distributions (15). Among the 10 genotypes, genotypes B and C are widespread in Asia, but two genotypes lead to distinctly different clinical outcomes (16). Compared to genotype B, genotype C showed high HBV replication capacity, with high levels of HBV DNA in the serum (17, 18). In addition, the tendency of chronicity was higher and more frequently developed into liver cirrhosis (LC) and hepatocellular carcinoma (HCC) in patients with genotype C than genotype B (19). However, the underlying mechanisms regarding distinct clinical and virological traits and distinct responses to IFN therapy between genotype C and genotype B remain unknown.

We recently introduced some mutations in the reverse transcriptase (RT) region of Pol related to HCC from genotype C infected patients [rtM80I, rtN139K/T/H, and rtM204I/V] (20). In addition to HBV mutations, it has been reported that there are several genotype dependent polymorphisms in HBV RT regions, which are generally defined as having a frequency <10% vs. wild type (Table S1). Of these, there are two polymorphisms at the Pol-269 site, rt269L (57.2%), and rt269I (42.8%), in patients with genotype C, in which two types are present with an almost similar ratio, but only rt269I is present as a major wild type in other genotypes.

In this study, we hypothesized that the presence of two HBV Pol RT polymorphisms distinct only in HBV genotype C may play a very pivotal role in viral phenotypes, clinical outcomes, and worse responses to IFN therapy distinct in genotype C infections. Thus, we sought to investigate the clinical factors and capacity to modulate IFN-I between two HBV polymorphisms, rt269L and rt269I, in genotype C infected CHB patients.

## Materials and Methods

### Patients

For this study, serum samples were collected from 410 patients chronically infected with HBV and the amino acid at 269th on RT region was identified by direct sequencing method. In 190 patients of these, their polymorphisms could not be identified by sequencing analysis due to the low sensitivity. However, 220 patients could be identified into their polymorphism, rt269L or rt269I types and selected for this study. To elucidate the correlation between the polymorphism of 269th amino acid and the characteristics of disease, their clinical factors and polymerase RT regions were assessed. This report was approved by the Institutional Review Board of Konkuk University Hospital (KUH-1010544) and Seoul National University Hospital (IRB-1808-067-965).

### HBV DNA Extraction and PCR Amplification for Polymerase RT Region

For this cohort study, HBV DNA was extracted from the serum of patients using a QIAamp DNA Blood Mini kit (QIAGEN, Hilden, Germany) and dissolved in Tris-EDTA buffer (10 mM Tris-HCl, 1 mM ethylenediaminetetraacetic acid, pH 8.0). First-round PCR was performed using primers POL-RT1 and the amplicon was used as a template for second-round PCR using primers POL-RT2 (Table S2). The PCR products were subject to direct sequencing analysis.

### HBV Genotyping

The 1,032-bp polymerase RT sequences were examined by direct sequencing and were compared to the sequences of the reference strains representing each of the genotypes (A-H including the C strains) obtained from GenBank. The sequences of the RT region were compared via Bayesian method for phylogenetic/molecular evolutionary analysis using MrBayes version 3.2.7, and the phylogenetic tree was constructed using FigTree version 1.4.3. Maximum-likelihood method was also used for the phylogenic analysis using MEGA version 10.0. Phylogenetic trees were reconstructed using 1,000 bootstrap replicates, and the mean genetic distances were estimated using Kimura two-parameter with Invariant sites and Gamma model.

### Plasmid and Site-Directed Mutagenesis

The pHBV-1.2x (GenBank accession No. AY641558) containing genotype C HBV full-length genome was used for site-directed mutagenesis to generate polymerase RT mutant DNA constructs using an *i*-pfu kit (iNtRON, Seongnam, Korea). Mutagenesis was performed using the primers rt269I based on rt269L construct. To exclude CMV promoter, HBV full genome constructs were cut by restriction enzyme *Sma*I and prepared for linear formed genome.

### Cell Culture and Transfection

Human hepatocellular carcinoma HepG2 cells and mouse hepatoma Hepa-1c1c7 purchased from the Korean Cell Line Bank (KCLB, Seoul, South Korea) were grown in minimum essential medium (MEM) supplemented with 10% fetal bovine serum (FBS), 100 μg/ml of penicillin-streptomycin, and 25 mM HEPES at 37°C in a humidified environment containing 5% CO_2_. pHBV-1.2x containing genotype C HBV full-length genome (2.5 μg) was transiently transfected using Lipofectamine 3000 (Invitrogen, Carlsbad, CA, USA). To normalize the transfection efficacy, pSV-β-Galactosidase (0.25 μg) was co-transfected, and the enzyme assay was accompanied using β-Galactosidase Enzyme Assay System with Reporter Lysis buffer (Promega, Madison, WI, USA), following the manufacturers' protocol.

### *In vivo* Assay and Hydrodynamic Injection

Female C57BL/6 mice (7 weeks old) were hydrodynamically tail-vein injected with HBV DNA (10 μg) per 8% of mouse body weight within 30 s. All of the animal experiments were approved by the Institutional Animal Care and Use Committee (IACUC) of Seoul National University College of Medicine (SNU-170308). Mice were sacrificed on 4, 7, and 16 days after HBV-encoding DNA injection, and liver and serum were collected to analyze gene expression of ISGs and HBV viral factor, respectively.

### Enzyme-Linked Immunosorbent Assay (ELISA)

To measure the HBV antigens in the blood serum or culture supernatant, ELISA was performed for HBsAg (Biokit, Barcelona, Spain) and HBeAg (AccuDiag, Oceanside, CA, USA) according to the manufacturers' instructions. The concentration of mouse and human IFN-β was measured with ELISA kit purchased from Biolegend (San Diego, CA, USA) and PBL assay Science (Piscataway, NJ, USA), respectively, according to the manufacturer's procedure.

### Covalently Closed Circular DNA (cccDNA) Extraction and Real-Time Polymerase Chain Reaction

The pHBV-1.2x with rt269L or rt269I were digested with restriction enzyme *Sma*I to remove CMV promoter, and the linear DNA (2.5 μg) were transfected using lipofectamine 3000 following manufacture's instruction. The transfected cells were lysed with lysis buffer (50 mM Tris-HCl, pH 7.4, l mM EDTA, and 1% NP-40), and the nuclei were collected via centrifugation and incubated with nucleus lysis buffer (10 mM Tris-10 mM Tris-HCl, 10 mM EDTA, 150 mM NaCl, 0.5% SDS, and 0.5 mg/ml protein K). The nucleic acids were purified via ethanol precipitation and treated with 10 U Plasmid-Safe ATD dependent DNase I (PSAD, Epicentre, Madison, WI, USA). The cccDNA was purified by PCI and ethanol precipitation and quantified via real-time PCR using SYBR and primers cccF and cccR (Table S2).

### Total RNA Extraction and Real-Time Polymerase Chain Reaction (qRT-PCR)

Total RNA was extracted from the transfected cells or mouse liver tissue using TRIzol, and the target genes were amplified using SensiFAST SYBR Lo-ROX One-Step kits (BioLine, London, UK). The transcription level was analyzed using qRT-PCR with sets of primers (Table S2) and housekeeping gene 18S ribosomal RNA was used as an internal control.

### IFN-I Luciferase Reporter Assay

Cell culture supernatants from transfected cells were overlaid on top of HEK293 IFN reporter cells containing ISRE-luciferase construct (21) and incubated for 4 h. The reporter cells were lysed in passive lysis buffer (Promega, Madison, WI, USA) for 30 min at room temperature, mixed with firefly luciferin substrate (Promega, Madison, WI, USA), and measured using an illuminometer (Beckman Coulter Inc., Fullerton, CA, USA).

### IFN-I Signal Block Assay

HepG2 cells were pre-treated with 10 μg of anti-IFNAR2 antibody (PBL assay Science, Piscataway, NJ, USA) and AZD1480 (Sigma, St. Louis, MO, USA) in MEM supplemented with 2% of FBS and transfected with pHBV-1.2x DNA constructs. STING-siRNA (Santa Cruz Biotechnology, Dallas, TX, USA) were co-transfected with pHBV-1.2x DNA constructs, following the manufacturers' procedures. Scrambled siRNA (Thermo-Fisher Scientific, Waltham, MA, USA) was used as control.

### Preparation of HBV From Transiently Transfected Cells and Infection Assay

For an infection study, culture supernatant of HepG2 cells, transiently transfected with 1.2x-rt269L or rt269I type of HBV plasmid, was collected. The supernatant was cleared through a sterile 0.45-μm pore size filter and precipitated with 6% polyethylene glycol (PEG) 8000 overnight. The media were ultracentrifuged and the collected pallet was resuspended in PBS containing 15–25% FCS. After quantification by qPCR, 3 × 10^9^ HBV genome equivalent per milliliter was aliquoted and stored at −80°C. HepaRG and HepG2-hNTCP-C4 cells were seeded in 12-well plates. Infection assay was performed with the concentrated virus in the presence of 4% PEG8000 at 37°C for 20 h. HepaRG was purchased from BIOPREDIC International (HPR116) and HepG2-hNTCP-C4 was kindly provided by Dr. Koichi Watashi (National Institute of Infectious Disease, Tokyo, Japan).

### Flow Cytometry and Confocal Analysis

The mtROS were stained with MitoSOX (5 μM), and analyzed by flow cytometry (LSRII, Becton Dickinson, San Jose, CA, USA) and confocal microscope (Confocal-A1, Nikon, Tokyo, Japan). DAPI was used to stain nuclei and the cells were mounted in mounting medium (VECTASHIELD Antifade Mounting Medium, H-1000). The images were captured using a 100 × oil immersion objective lens.

### 8-OHdG ELISA Assay

Genomic DNA was extracted from the transfected cells using QIAamp Blood DNA extraction kit (QIAGEN, Hilden, Germany). For the detection of 8-hydroxy-2′-deoxyguanosine (8-OHdG) activity, a competitive ELISA for 8-OHdG analysis kit (OxiSelect Oxidative DNA Damage ELISA kit, Cell Biolabs, San Diego, CA, USA) was used according to the manufacture's protocol.

### Measurement of NO${}_{2}^{-}$ and NO${}_{3}^{-}$ Levels

The produced NO was measured in culture supernatants obtained from the transfected cells using a colorimetric Nitrite/Nitrate Assay Kit (Sigma, St. Louis, MO, USA) according to the manufacturer's instructions, and measured the absorbance at 540 nm in microplate reader (Tecan Infinite M200 Pro, Tecan Group Ltd., Männedorf, Switzerland).

### Statistical Analyses

For the cohort study, statistical analysis was performed using SPSS Software (IBM SPSS version 23.0.0.0 Inc., Chicago, USA). Categorical variables were analyzed using Multivariate Analysis of Variance (MANOVA). Independent *t*-test was used to compare continuous variables. Tests were two-sided.

Experimental data were analyzed using Graphpad Prims 5 (GraphPad Software, La Jolla, CA, USA). All experiments were independently repeated three times and the statistical analyses were indicated in the figure legends. The *p*-value of statistical significance was set at either; ^*^p < 0.05; ^**^p < 0.01; ^***^p < 0.001.

## Results

### rt269I Was Related to Enhanced Disease Progression in a Korean Cohort With Genotype C Infections

In the web page “HBV RT: Mutation prevalence according to genotype and treatment” (https://hivdb.stanford.edu/HBV/DB/cgi-bin/MutPrevByGenotypeRxHBV.cgi.), the mutation prevalence or polymorphisms of the entire HBV RT region were analyzed according to the genotypes and antiviral drug treatments (Table S1). A comparison of all of the 344 codons of RT found one unique genotype C site at the 269th codon of RT, in which both types, one for rt269L encoding leucine and the other for rt269I encoding isoleucine, were present in patients infected with genotype C at frequencies of 57.2 and 42.8%, respectively. Although rt269 codon is located at the outer region of overlapped HBsAg, polymorphism of rt269 cannot lead to HBsAg amino acid change, not affecting the performance of the HBsAg kit or immunoblotting data. There were no other polymorphisms in patients infected with genotype C exceeding 10% of the total frequency. Therefore, we postulated that the polymorphism at the 269th codon of RT may play an important role in the clinical outcomes and pathogenesis of genotype C infections. To address this issue, we analyzed the 269th codon polymorphisms of 220 patients in our Korean cohort with genotype C via direct sequencing of the RT region (Figure S1) and compared their clinical factors between rt269L and rt269I. Overall, 138 patients (62.7%) and 82 patients (37.3%) were infected with rt269L or rt269I, respectively (Table 1). We also found there were several patients mixed with both rt269L and rt269I in sequencing data, maybe due to quasi species generation. In these cases, we determined their polymorphisms types into one reflecting dominance in sequencing data.

**Table 1:** Comparison of the clinical features between patients infected with the two types of genotype C, rt269L, and rt269I.

**Clinical factor**	**rt269L (*n* = 138)**	**rt269I (*n* = 82)**	***p*-value**
CH:HCC	92:46	46:36	ns
Sex, men	63.04%	64.63%	ns
HBeAg negative	28.99%	52.44%	^**^
AST/ALT	1.16 ± 0.78	1.42 ± 1.43	ns
HBV-DNA (log10 copies/ml)	6.47 ± 1.71	5.75 ± 1.62	^**^
HBsAg (log10 IU/ml)	3.74 ± 0.61	3.41 ± 0.71	^***^
Age (years)	42.04 ± 12.45	45.89 ± 12.61	^*^
Bilirubin (mg/dL)	0.99 ± 0.91	1.37 ± 1.71	^**^
Albumin (g/dL)	4.01 ± 0.58	3.95 ± 0.63	ns
Prothrombin time (INR)	1.11 ± 0.18	1.14 ± 0.18	ns
Platelet (10^9^/L)	169.93 ± 63.74	168.43 ± 81.93	ns
Fibrosis-4 score	4.05 ± 7.96	4.45 ± 5.42	ns
**Disease phase^†^**			^***^
1	18.12%	6.10%	
2	52.90%	41.46%	
3	24.64%	43.90%	
4	4.35%	8.54%	

*CH, chronic hepatitis; HCC, hepatocellular carcinoma; AST, aspartate aminotransferase; ALT, alanine transaminase*.

† *1. Immune-tolerant. 2. Immune-clearance. 3. HBeAg negative CHB. 4. Inactive carrier. Multi variate test were used*.

Continuous variables were tested using the independent t-test and categorical variables were analyzed using MANOVA. ns, non-significant;

* p < 0.05;

** p < 0.01;

*** *p < 0.001*.

Interestingly, there were distinct disparities in some clinical factors between the patients with rt269L and rt269I (Table 1). The patients with rt269I had significantly lower levels of HBV DNA and HBsAg, HBeAg negative infection, and liver disease progression based on immune response against HBV (22). Our data suggest that both rt269L and rt269I on the RT region may be key factors determining clinical outcomes regarding disease progression and HBeAg negative infection in genotype C infected patients.

### The Higher Frequency of preC Mutation (G1896A) in Patients With rt269I Infections Is Responsible for the Higher Frequency of HBeAg Negative Infections

Of the various HBV mutations, G1896A mutations on the pre-core region (preC mutation) and A1762T/G1764A double mutations on the basal core promoter (BCP) lead to HBeAg negative infection that are significantly related to liver disease progression (23). To verify whether these mutations are associated with distinct clinical factors of genotype C infections between rt269L and rt269I, their mutation rates were compared not only in chronic infectious patients in our cohort study, but also in the reference strains in GenBank (Table 2). In double mutations in BCP, only the G1764A mutation, but not the A1762T, was significantly prevalent in patients with rt269I of both our cohort and GenBank. In addition, the rate of G1986A preC mutation was significant higher in patients with rt269I (49.61%) than those with rt269L (28.83%, *p* < 0.01). These results suggest that the high frequency of G1986A preC mutation is responsible for HBeAg negative infections in patients with genotype C rt269I infections.

**Table 2:** The frequencies of BCP and preC mutations between rt269L and rt269I in a Korean cohort and from reference strains.

**Mutation**		**Nucleotide**	**Clinical isolates**			**Reference strains**		
			**rt269L (*n* = 115)**	**rt269I (*n* = 69)**	***p*-value**	**rt269L (*n* = 111)**	**rt269I (*n* = 129)**	***p*-value**
BCP mutation	A1762T	A	47 (40.87%)	23 (33.33%)	ns	31(27.93%)	24 (18.60%)	ns
		T	68 (59.13%)	46 (66.67%)		80(72.07%)	105 (81.40%)	
	G1764A	G	44 (38.26%)	16 (23.19%)	^*^	29(26.13%)	17 (13.18%)	^*^
		A	71 (61.73%)	53 (76.81%)		82(73.87%)	112 (86.82%)	
preC mutation	G1896A	G	83 (72.17%)	33 (47.83%)	^***^	79(71.17%)	65 (50.39%)	^***^
		A	32 (27.83%)	36 (52.17%)		32(28.83%)	64 (49.61%)	

*BCP, basal core promoter; preC, pre-core*.

Continuous variables were tested using the chi-squared test. ns, non-significant;

* p < 0.05; **p < 0.01;

*** *p < 0.001*.

### rt269I Led to Lower Levels of HBV Replication in *in vitro* and *in vivo* Experiments

**Figure 1:**
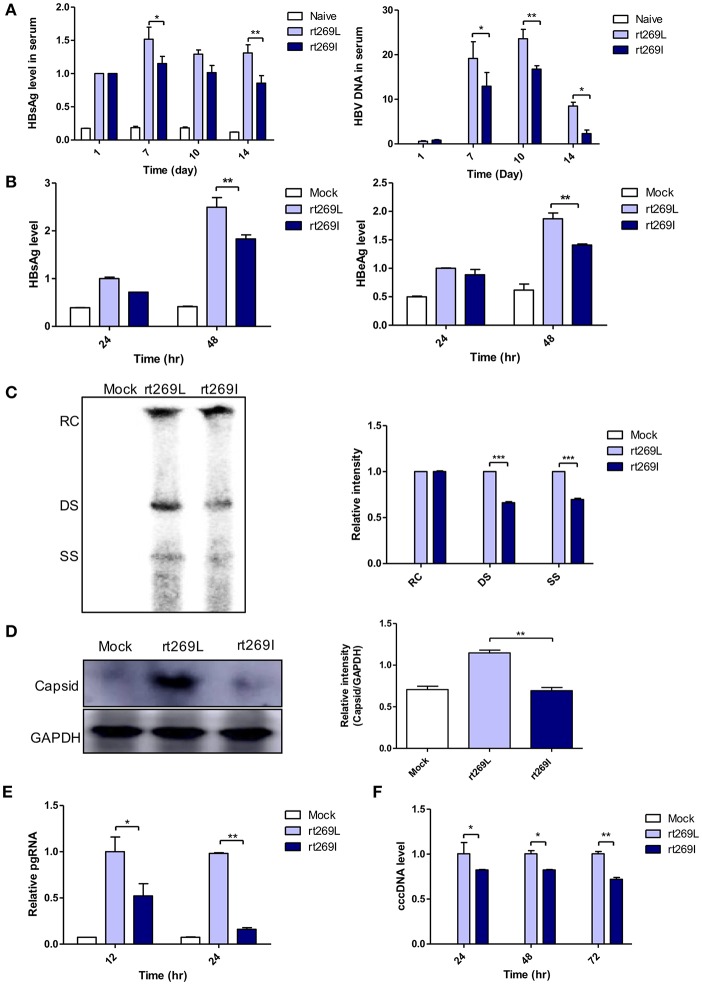
rt269I vs. rt269L led to reduced viral replication in *in vivo* and *in vitro* analyses. **(A)** Both HBV-encoding DNA and pSV-β-Galactosidase was injected by hydrodynamic tail vein injection into C57BL/6 mice. HBsAg and HBV DNA in serum were determined by HBsAg ELISA and normalized with β*-Galactosidase* (*n* = 5). **(B–E)** Plasmid DNA was transfected into HepG2 cells and the replicative capacities were analyzed. **(B)** The level of secreted HBsAg and HBeAg were detected using ELISA. **(C)** HBV DNA was compared via Southern blotting at 48 h post-transfection. **(D)** Western blot analyses of intracellular capsid forms were performed by using anti-HBcAg antibody on naïve gel at 24 h after transfection. **(E)** The translation mediates pgRNAs were detected by qRT-PCR. **(F)** Linear DNA removed from CMV promoter was transiently transfected into HepG2 cells. The intermediates of transcripts cccDNA were determined using qPCR. Data were normalized to β-Galactosidase enzyme assay. One- and two-way ANOVA were used. ^*^p < 0.05, ^**^p < 0.01, ^***^p < 0.001.

Our clinical and epidemiologic data suggest there may be differences in the HBV replication capacity between rt269I and rt269L infections. To verify this hypothesis, we performed *in vivo* and *in vitro* study with genotype C HBV full genome constructs with leucine or isoleucine at the 269th amino acid on the RT region. HBV replications between rt269I and rt269L infections were analyzed via *in vivo* and *in vitro* studies. The secreted HBsAg and HBV DNA were greatly increased in the serum from the mice infected with rt269L. However, rt269I showed relatively lower HBV replication levels (Figure 1A). These replicative capacities were also examined in HepG2 cells via transient transfection of the HBV constructs and the linear DNA constructs (Figure S2A), in which CMV promoter was deleted (24). As shown in the *in vivo* study, the secreted HBsAg and HBeAg levels also decreased in HepG2 cells, Huh-7, or Huh-7.5 cells transfected with rt269I (Figure 1B and Figures S2B,C). Intracellular intermediates of HBV amplification were also analyzed via Southern blotting, which indicated that double-stranded (DS) and single-stranded (SS) DNA were significantly reduced in rt269I at 48 h post-transfection (Figure 1C). In addition, the HBV capsid form that is an intermediate of HBV amplification was significantly reduced in rt269I at 24 h after transfection (Figure 1D). Similarly, rt269I also showed decreased pgRNA levels and cccDNA levels (Figures 1E,F).

### rt269I Led to Enhanced IFN-I Signaling

**Figure 2:**
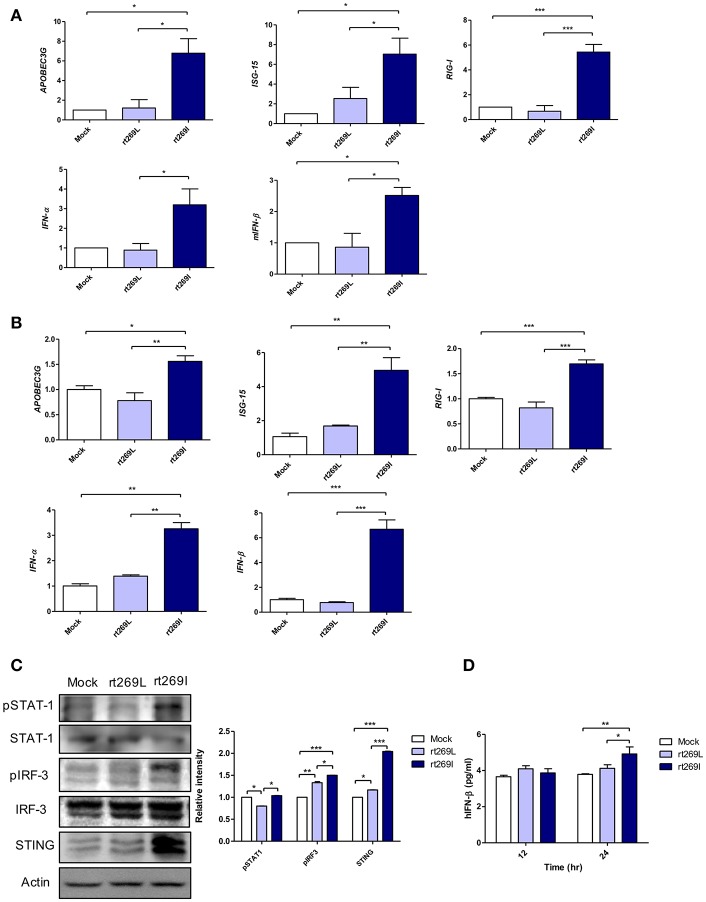
rt269I enhanced IFN-I production in HepG2 cells. **(A)** Both HBV-encoding DNA and pSV-β-Galactosidase were injected by hydrodynamic tail vein injection into C57BL/6 mice (*n* = 5). The transcription level of APOBEC3G, ISG-15, RIG-I, IFN-α, and IFN-β were determined by qRT-PCR from mice hepatocytes on 4 days post-infection, and normalized with 18S rRNA and β*-Galactosidase*. **(B–D)**. HepG2 cells were transfected with HBV DNA and the expression levels of IFN-I-related genes were assessed. **(B)** The mRNA level of IFN-I-related genes was determined using qRT-PCR at 12 h after transfection, and data were normalized to 18S rRNA. **(C)** The upstream proteins of the IFN-I signaling pathway were detected via Western blotting at 48 h post-transfection. **(D)** The secreted IFN-β was measured with ELISA at 24 h post-transfection. Data were and normalized to β-Galactosidase assay. One- and two-way ANOVA were used. ^*^p < 0.05, ^**^p < 0.01, ^***^p < 0.001.

Since IFN-I mediated antiviral effects by up-regulating APOBEC3G that hypermutate the HBV genome (25), we postulated that rt269I could enhance IFN-I mediated APOBEC3G signaling in infected hepatocytes and finally lead to the higher frequency of G to A mutations on preC and BCP in patients with rt269I. To address this issue, we compared the gene expressions of two representative IFN-I genes, INF-α and INF-β, and interferon stimulated genes, ISG-15, RIG-I, and APOBEC3G that could be induced by an IFN-I signal (26). The mRNA levels of APOBEC3G, ISG-15, RIG-I, IFN-α, and IFN-β were significantly enhanced in the mice infected with rt269I on 4 days post-infection (Figure 2A). In the same manner as shown in *in vivo* assays, the transcription levels of APOBEC3G and IFN-I-related genes, such as ISG-15, RIG-I, IFN-α, and IFN-β, were also induced in HepG2 and Huh7 cells transfected with rt269I at 12 h after transfection (Figure 2B and Figure S3). To evaluate the upstream genes of the IFN-I signaling pathway, the activation or expression of STAT-1, IRF-3 and the STING genes (27, 28) were analyzed using Western blotting at 48 h post-transfection (Figure 2C). rt269I activated STAT-1, IRF-3 and up-regulated STING in protein level. The secreted IFN- β was also assessed by ELISA and treating culture supernatant from the transfected cell onto HEK293 with luciferase construct under an interferon stimulated response element (ISRE) promoter (21). As the results of activated IFN-I signal, the secreted IFN-I was significantly induced in cells transfected with rt269I at 24 h post-transfection (Figure 2D and Figure S4A). These results were consequently shown in the Huh-7 or Huh-7.5 cells defective in RIG-I signaling at 24 h after transfection (Figures S4B,C), suggesting that the enhancing effect of IFN-I found in rt269I may be due to pathways other than the RIG-I dependent pathway. Notably, rt269I showed enhanced IFN-I, even compared to genotype A wild type with the same isoleucine at the 269th amino acid of RT at 24 h after transfection (Figure S4D), suggesting that the trait capable of IFN-I induction as found in rt269I may be specific to genotype C. In addition, the HBV encoding DNA was also transfected into mouse hepatoma cell Hepa1c1c-7 and IFN-β was measured assessed by ELISA (Figure S4E). Consistent with human hepatocellular cells, mouse hepatoma transfected with rt269I vs. rt269L produced enhanced IFN-β productions. Together, our data indicated that rt269I led to enhanced IFN-I production in hepatocytes.

### The Reduced Replication Capacity and Enhanced IFN-I Expression of rt269I vs. rt269L Were Also Proved in Two HBV Infection Models, HepaRG and HepG2-hNTCP-C4 Cells

**Figure 3:**
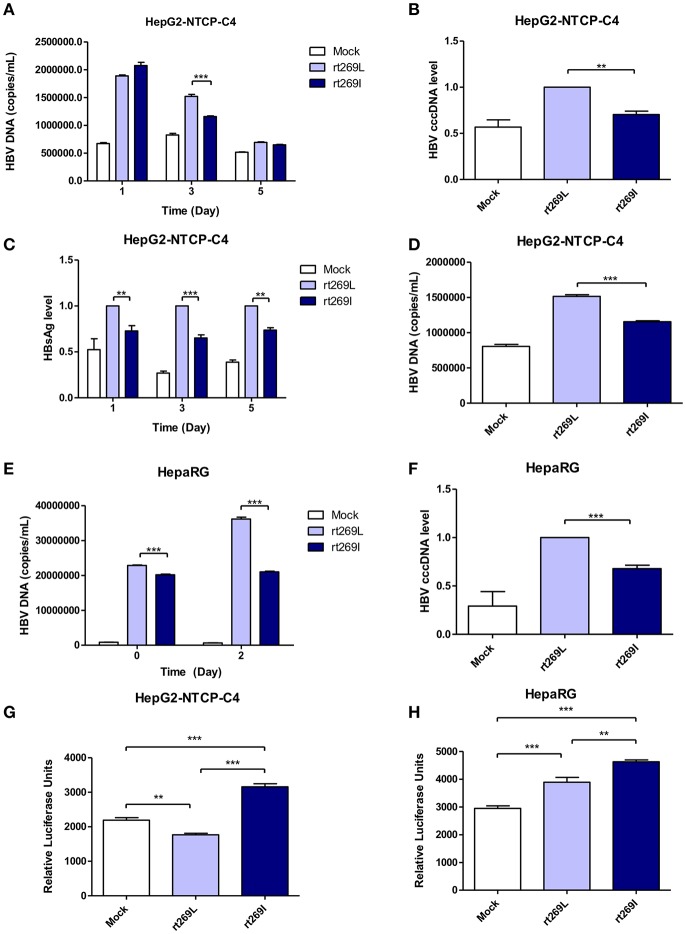
The enhanced replication capacity of rt269L vs. rt269I shown in HBV infection models **(A–D)** rt269L- or rt269I- HBV variant infection of HepG2-hNTCP-C4 cells. **(A)** Intracellular HBV DNA level were evaluated using qPCR on day 1, 3, and 5. **(B)** HBV cccDNA level was measured in HepG2-hNTCP-C4 cells infected by each HBV variant on day 5. **(C)** HBsAg secretion in the supernatant of infected cells were measured using ELISA on day 1, 3, and 5. **(D)** Extracellular HBV DNA level were evaluated using qPCR on day 3. **(E,F)** rt269L- or rt269I- HBV variant infection of HepaRG cells. **(E)** Intracellular HBV DNA level was measured using qPCR on day 0 and 2. **(F)** HBV cccDNA level was evaluated in HepaRG cells infected by each HBV variant on day3. **(G,H)** The secreted IFN-I in HepG2-hNTCP-C4 **(G)** and HepaRG cells **(H)** infected by each construct was measured using luciferase reporter assay on Day 5 and day 0, respectively. Data represents mean ± S.D. of three independent experiments. One- and two-way ANOVA were used. ^*^p < 0.05, ^**^p < 0.01, ^***^p < 0.001.

To determine whether the different replication capacity between rt269L and rt269I could be reproduced in HBV virion infection assays, we used two cell lines, HepaRG and HepG2-hNTCP-C4 which have been widely used for the HBV infection models (29, 30). In HepG2-hNTCP-C4 cells, intracellular HBV DNA level was also significantly increased in rt269L HBV infected cells compared with that of rt269I HBV infected cells on day 3 (Figure 3A). In addition, rt269I-HBV-infected HepG2-hNTCP-C4 cells showed lower cccDNA level compared with rt269L-HBV-infected cells on day 5 (Figure 3B). Furthermore, the secreted HBsAg was significantly elevated in rt269L vs. rt269I HBV infected group and this pattern was maintained until day 5 after infection (Figure 3C). Consistently, extracellular HBV DNA level was also increased in rt269L HBV infected cells compared with that of rt269I HBV infected cells on day 3 (Figure 3D). The different replication capacities from infection between rt269L and rt269I HBV were further verified in HepaRG cells. Intracellular HBV DNA level was greatly increased in rt269L vs. rt269I HBV infected group, and the most distinct difference between two groups was shown on day 2 (Figure 3E). Reduced cccDNA level, shown in rt269I-HBV infected HepG2-hNTCP-C4 cells, was also reproduced in HepaRG cells on day 3 (Figure 3F). Furthermore, IFN-I secretion levels were measured by an ISRE promoter-luciferase assay. As shown in transient transfection system, rt269I-infected-HepG2-hNTCP-C4 cells and –HepaRG cells secreted larger amount of IFN-I compared with rt269L-infected cells on Day 5 and day 0, respectively (Figures 3G,H). Together, we also proved that rt269I vs. rt269L could lead to reduced HBV replication and enhanced IFN-I production in HBV virion infection model system.

### The Replication of HBV rt269I Was Inhibited via STING- IFN-I Axis

**Figure 4:**
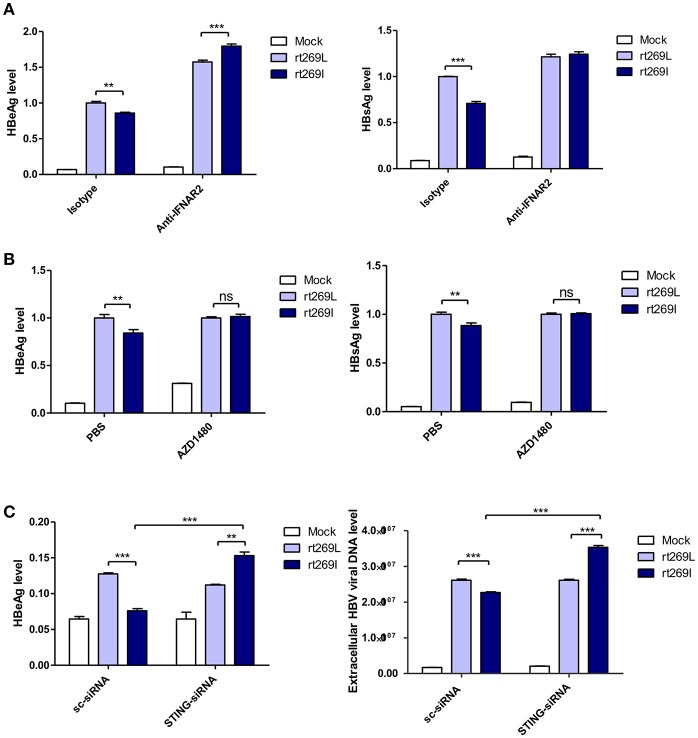
The replication inhibition found in rt269I was mediated via STING-IFN-I axis. **(A)** IFNAR2 was blocked using anti-IFNAR2 antibody (10 μg) and HBV-encoding DNA and pSV-β-Galactosidase were transiently transfected into HepG2 cells. HBsAg and HBeAg in the presence of isotype or anti-IFNAR2 antibody were measured using ELISA at 48 h post-transfection. **(B)** HepG2 cells pre-treated with AZD1480 (5 μM) for 2 h were transfected with HBV DNA pSV-β-Galactosidase and constantly treated with AZD1480. HBsAg and HBeAg were measured using ELISA at 48 h post-transfection. **(C)** HBV DNA constructs and pSV-β-Galactosidase were co-transfected with either STING or scramble siRNA into HepG2 cells. The HBeAg levels and HBV virions were analyzed using ELISA and qPCR, respectively, at 48 h post-transfection. Data were normalized with β-Galactosidase enzyme assay, and represents mean ± S.D. of three independent experiments. One- and two-way ANOVA were used. ^*^p < 0.05, ^**^p < 0.01, ^***^p < 0.001.

It has been reported that the secretion of IFN-I exerts antiviral effects against HBV (31, 32), and our above finding also showed that IFN-I was produced in the cells transfected with rt269I. Therefore, we verified whether replication inhibition found in rt269I is dependent on IFN-I signaling. We found that the decreased HBsAg and HBeAg levels in rt269I were neutralized or even reversed when the IFN-I signal was blocked via IFNAR2 neutralization at 48 h post-transfection (Figure 4A). To further check IFN-I signal dependence of rt269I replication, JAK-STAT pathway was inhibited using AZD1480. As shown in IFNAR2 neutralization, the inhibition of HBsAg and HBeAg level in rt269I was reversed by inhibition of JAK-STAT pathway at 48 h after transfection (Figure 4B). Furthermore, we found that the siRNA mediated knockdown of STING, but not scramble could lead to reversion of inhibition on HBV replication or HBsAg secretion as found in the rt269I type at 48 h post-transfection (Figure 4C). Together, these results indicated that rt269I led to lower levels of HBV replication via STING- IFN-I-axis in hepatocytes.

### rt269I Led to Mitochondrial Stress Mediated Enhanced IFN-I Production and Heme Oxygenase (HO)-1

**Figure 5:**
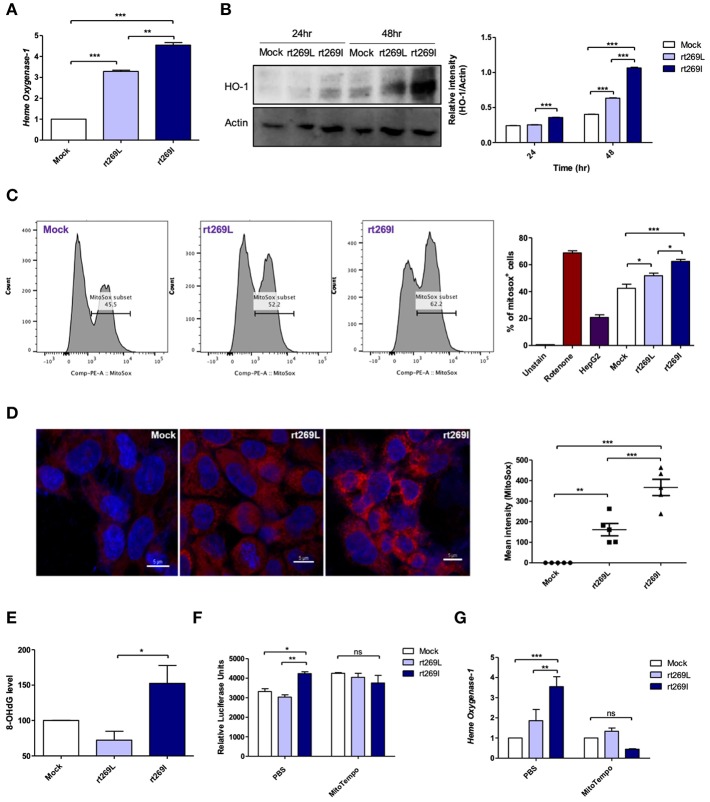
rt269I led to enhanced HO-1 production and mitochondrial stress in infected hepatocytes. Mitochondrial stress was assessed from HepG2 cells transfected with HBV DNA. pSV-β-Galactosidase was co-transfected and used to normalize β-Galactosidase assay **(A)** Transcription level of HO-1 was determined using qRT-PCR at 24 h after transfection. **(B)** HO-1 protein was detected via Western blotting and actin was used as a control. **(C,D)** Mitochondrial ROS was stained with MitoSOX (5 μM) and assessed by flow cytomery **(C)** and confocal microscope **(D)**, followed by transfection for 24 h. **(E)** 8-OHdG ELISA was performed with genomic DNA at 48 h post-transfection. **(F,G)** MitoTEMPO (100 μM) was administrated to the cells. **(F)** IFN-I levels were assessed using an IRSE-luciferase assay at 24 h after transfection. **(G)** qRT-PCR analysis of HO-1 mRNA levels were evaluated using qRT-PCR at 24 h after transfection. Data represent mean ± S.D. of three independent experiments. ^*^p < 0.05, ^**^p < 0.01, ^***^p < 0.001.

As bilirubin is one of the final byproducts of inducible HO-1 (33), our clinical data showing higher levels of bilirubin in serum of patients with rt269I (Table 1) also suggest that rt269I could enhance HO-1 expression. As expected, the expressed HO-1 significantly increased in the transcription and translation levels in cells with rt269I (Figures 5A,B). Previously, HO-1 has been reported to be induced by mitochondrial stress or ROS (34). Thus, we assessed whether mitochondrial ROS (mtROS) could be enhanced in HepG2 cells infected with rt269I. Although both groups showed similar cytosolic ROS levels (data not shown), enhanced mtROS levels were found in cells with rt269I at 24 h post-transfection (Figures 5C,D), suggesting that rt269I vs. rt269L led to enhanced mitochondrial stress. To assess whether or not this effect is specific to genotype C, we compared mtROS production in HepG2 cells between two types of genotype C, rt269L and rt269I, and the wild type of genotype A with Pol of rt269I type via confocal analysis using MitoSOX. Of these, genotype C-rt269I produced the strongest mtROS, suggesting that the enhancing trait of mtROS found in rt269I may also be specific to genotype C at 24 h after transfection (Figure S5). It has been reported that mtROS can cause oxidative mitochondrial DNA damage, resulting in increased mitochondrial 8-OHdG that is indicative of DNA damage (35). We consistently observed that the quantification of 8-OHdG significantly increased in cells infected with rt269I at 48 h post-transfection (Figure 5E). Furthermore, we assessed if mtROS could act as upstream signaling of enhanced IFN-I and HO-1 induction induced by rt269I via treatment of MitoTEMPO, a mitochondria-targeted antioxidant. The treatment of MitoTEMPO abrogated the increased IFN-I induced by rt269I at 24 h post-transfection (Figure 5F). In addition, the elevated HO-1 mRNA levels induced by rt269I were also completely abrogated by MitoTEMPO treatment at 24 h post-transfection (Figure 5G). Together, these results indicated that rt269I infections could lead to mitochondrial stress and the subsequent cytosol release of oxidized mtDNA, resulting in enhanced production of IFN-I and HO-1 in hepatocytes.

### rt269I Led to Enhanced iNOS Dependent NO Production

**Figure 6:**
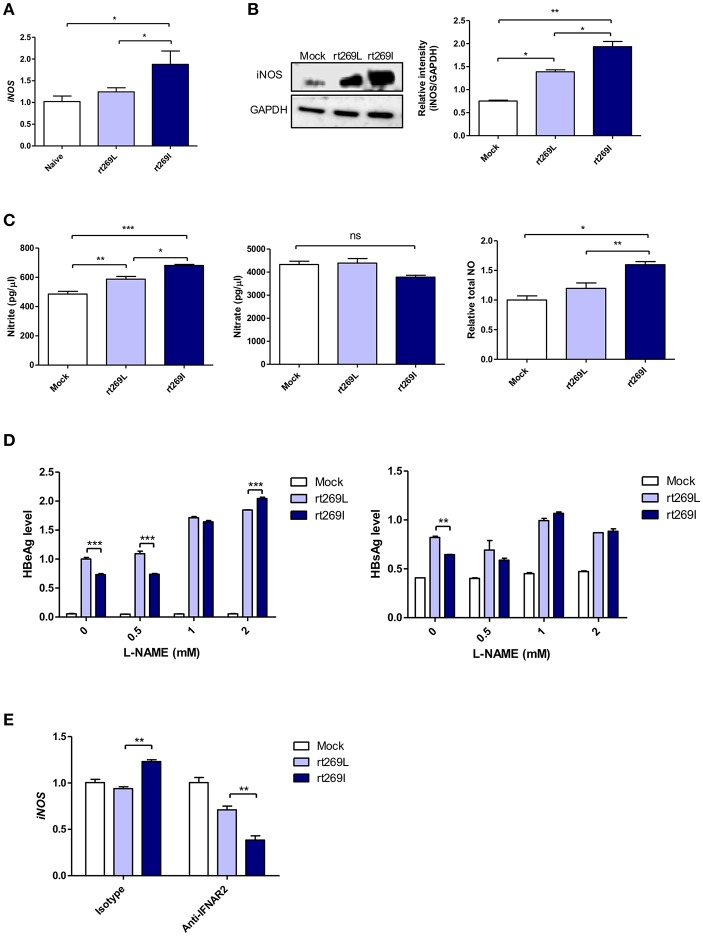
rt269I enhanced iNOS dependent NO production in an IFN-I dependent manner. **(A)** The HBV constructs were injected with hydrodynamic tail vein into C57BL/6 mice (*n* = 5). Transcription levels of iNOS in hepatocytes were determined using qPCR and were normalized with 18S rRNA on 3 days post-injection. **(B–E)** HepG2 cells were transfected with HBV construct and pSV-β-Galactosidase was co-transfected to normalization. **(B)** iNOS synthesis was detected via Western blot analysis at 48 h after transfection. **(C)** Poly(I:C) was co-transfected with HBV DNA and the secreted NO${}_{2}^{-}$ and NO${}_{3}^{-}$ were measured using ELISA at 24 h post-transfection. **(D)** HepG2 cells pre-treated with L-NAME for 2 h were transfected with HBV DNA and constantly treated with L-NAME. The secreted HBsAg and HBeAg were detected by ELISA at 24 h. **(E)** IFN-I signaling was blocked by anti-IFNAR2 antibody (5 μg) for 12 h and iNOS gene expression was determined using qRT-PCR at 24 h after transfection. Data are shown as mean ± SD. One- and two-way ANOVA were used. ^*^p < 0.05; ^**^p < 0.01; ^***^p < 0.001; ns, not significant.

It was previously reported that HBV infections could lead to nitric oxide (NO) mediated by inducible nitric oxide synthase (iNOS), which causes the inhibition of viral replication (36). Thus, we assessed iNOS dependent NO production between rt269I and rt269L. In an *in vivo* mouse model, the transcription of iNOS was significantly enhanced in rt269I compared to rt269L on 3 days post-injection (Figure 6A). In *in vitro* systems of transfected HepG2 cells, rt269I led to enhanced iNOS expression via Western blotting at 48 h post-transfection (Figure 6B). The secreted NO measured using ELISA was also significantly increased in rt269I at 24 h post-transfection (Figure 6C). In addition, to better understand the antiviral effects of NO, the viral replication of both types was also assessed after treatment with iNOS inhibitor, N^ω^-Nitro-L-arginine methyl ester hydrochloride (L-NAME). Treatment with L-NAME restored decreased HBsAg and HBeAg levels in both rt269I and rt269L types, but its effect was more pronounced in rt269I, suggesting that the lowered replication shown in rt269I may be in part due to its enhanced NO production at 24 h post-transfection (Figure 6D). It has been reported that IFN-I can regulate iNOS dependent NO production as its upstream signal (37). To further verify whether iNOS induction found in rt269I is dependent on IFN-I signaling, the iNOS expression levels of both types were analyzed from infected HepG2 cells after blocking IFNAR2. The increased transcription levels of iNOS found in rt269I were abrogated by blocking IFNAR2 at 24 h post-transfection (Figure 6E). Together, our data indicated that rt269I could lead to enhanced iNOS dependent NO production via IFN-I signaling, contributing to disease progression in chronic patients with genotype C infections.

## Discussion

Of 10 different HBV genotypes, genotype C and genotype B are responsible for the majority of HBV infections in endemic Asian countries (17). In particular, it has been reported that most HBV infections in South Korea are due to genotype C (38), which may be a major reason for the lower response of IFN therapy (39) or NA therapy (40), the higher level of disease progression (41), and the higher prevalence of occult infection via vertical transmission (42, 43), observed in Korean CHB patients compared to patients in other areas. The current study investigated the virological or clinical factors and modulating capacity of antiviral IFN-I innate immunity between rt269L and rt269I in HBV genotype C infections. There are some noteworthy findings.

First, our clinical data using a Korean cohort proved that rt269L and rt269I showed distinct clinical or virological traits in HBV replication, HBsAg production, HBeAg serostatus, and biliverdin production (Table 1). This strongly suggests that there may be differences in modulating antiviral IFN-I production between rt269L and rt269I. Given a previous report indicating that the better response of genotype B than that of genotype C infections in IFN-α therapy was attributed to a higher level of HBeAg seroconversion via IFN-α mediated preC mutations (44), rt269I vs. rt269L, more related to lower HBV replication or the HBsAg level and more related to HBeAg negative serostatus was expected to lead to enhanced IFN-I production. Furthermore, a higher level of bilirubin, which is one of the final products of IFN-I mediated HO-1 metabolism, was also found in patients with rt269I type, further supporting the aforementioned findings.

Second, our further epidemiologic data showed that HBeAg seronegative status observed in patients with rt269I was due to a higher frequency of preC mutations (G to A at 1896) induced via IFN-I mediated APOBEC3G (Table 2 and Figure 2). This suggests that longer durations of HBeAg positive stages and lower levels of preC mutation frequency observed in patients with genotype C than that of genotype B infections (17) may be due to the presence of rt269L only in patients with genotype C. Actually, our further mechanism study showed that rt269L could produce lower levels of IFN-I production than rt269I, leading to a lower expression of ISGs including APOBEC3G in *in vitro* and *in vivo* systems, further supporting our epidemiological findings. Our epidemiologic data also showed that rt269L (63%) was more prevalent than rt269I (37%) in our Korean cohort, unlike other areas showing more prevalence of rt269I vs. rt269L (rt269I vs. rt269L: 57 vs. 43%). This suggests that rt269L showed not only high replication factors such as HBeAg, HBsAg, and HBV DNA level but also low preC mutation frequencies. These features of rt269L can be indicated with characteristics of genotype C, which was not shown in other genotypes. In addition, we suggest it may have a merit in infections into hosts via perinatal or vertical routes, contributing to chronic infections as a major type in South Korea. It is tempting to speculate that the higher prevalence of rt269L may contribute to some unique clinical traits found in South Korean CHB patients, including the frequent failure of IFN-α therapy or NA treatments (39, 40) and the higher prevalence of occult HBV infections via vertical routes (42, 43). However, this issue demands further investigation in the future.

Third, our mechanism study indicated that distinct IFN-I production between rt269L and rt269I was attributed to different induction capacity of mitochondrial stress (Figure 5). Thus, rt269I vs. rt269L can lead to enhanced production of mtROS and oxidized mtDNA, resulting in induced IFN-I production via cytosolic DNA sensing through the STING-IRF3 dependent pathway. To date, distinct capacity of mitochondrial stress induced by HBV genotypes, polymorphisms, or mutations has not been elucidated. HBV Pol was recently reported to interfere with antiviral IFN-I production through DNA sensing via the inhibition of STING polyubiquitination (13). Thus, the functional difference in Pol due to SNP could contribute to differences in the two types of mitochondrial stress mediated IFN-I production. However, the molecular details regarding this issue remain to be elucidated in the future.

**Figure 7:**
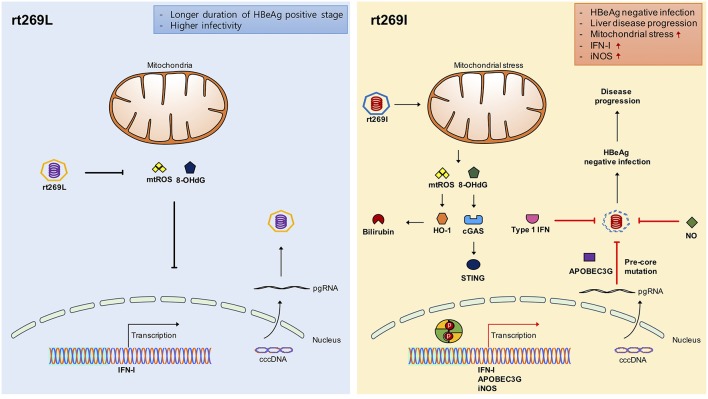
Schematic presentation indicating distinct mitochondrial stress mediated IFN-I production and its distinct contribution to disease progression in chronic patients with genotype C infections between rt269L and rt269I. In contrast to rt269L, rt269I infection in genotype C induced mitochondrial ROS production, which led to an increased release of oxidized DNA into the cytosols of the infected hepatocytes. Sensing the oxidized DNA exposed to cytosol via the cGAS-STING pathway could lead to IFN-I production. Enhanced INF-I production in rt269I could exert several biological activities. First, it can lead to the increased inhibition of HBV replication via the inhibition of capsid formation by HO-1 production. Second, IFN-I mediated enhanced expression of APOBEC3G and iNOS can lead to HBeAg negative infection and liver disease progression via frequent generation of preC mutations at 1896 (G to A). Thus, rt269L can contribute to HBeAg negative infection and disease progression in chronic patients infected with genotype C via mitochondrial stress mediated enhanced INF-I production.

Fourth, we found enhanced HO-1 expression and iNOS dependent NO production as a downstream pathway of IFN-I in rt269I. The enhanced HO-1 expression in rt269I exerted anti-HBV effects via the inhibition of virion capsid formation, in line with previous reports showing the anti-HBV effects of HO-1 (45). It has also been reported that iNOS dependent NO production as another downstream pathway of INF-I could play a pivotal role in inflammatory responses and disease progression in the liver, including fibrosis and cirrhosis (37). We also found enhanced production of iNOS and NO in HepG2 cells transfected with rt269I (Figure 6), in line with our clinical data showing that patients with rt269I type infections were more related to disease progression. Enhanced iNOS and NO could also increase the frequency of preC mutations shown in rt269I, leading to HBeAg negative infections in chronic patients. The iNOS dependent NO production and mtROS production can synergistically exert apoptotic cell death in the liver, also facilitating the progression of liver diseases (46), further supporting our hypothesis regarding the contribution of rt269I to disease progression (Figure 7).

Fifth, our finding showing rt269L vs. rt269I type led to enhanced HBV replication via inhibiting IFN-I production via STING-IFN-I axis is contrast to that reported by Ahn et al (47), previously. The difference between the two studies may be due to the use of HBV genome plasmid from different patients with distinct genome sequences or difference of used cell lines. The pHBV-1.2x (GenBank accession No. AY641558) plasmid construct used in this study has no special mutations affecting HBV virology and virulence and have been widely used for genotype C mutation analysis and virulence studies (24, 48–50). Furthermore, together with our HBV genome transient study into diverse human hepatocytes and mouse hepatoma cell lines and *in vitro* HBV virion infection study, our *in vivo* hydrodynamic injection study, and even our clinical data consistently supported enhanced HBV replication of rt269L vs. rt269I type.

In conclusion, our data showed that rt269I could contribute to HBeAg negative infections and liver disease progression in CHB patients with genotype C infections via mitochondrial stress mediated IFN-I production. In addition, enhanced iNOS dependent NO production induced by rt269I could also provide an additive role in disease progression. Furthermore, our findings could also provide a likely explanation into characteristic features of genotype C with higher frequency of rt269L type, including longer durations of HBeAg positive stages and higher infectivity (Figure 7).

## Data Availability

The datasets generated for this study are available on request to the corresponding author.

## Ethics Statement

This report was approved by the Institutional Review Board of Konkuk University Hospital (KUH-1010544) and Seoul National University Hospital (IRB-1808-067-965). All of the animal experiments were approved by the Institutional Animal Care and Use Committee (IACUC) of Seoul National University College of Medicine (SNU-170308).

## Author Contributions

S-YL and B-JK designed the study. S-YL performed statistical analysis. S-YL, Y-MC, and S-JO performed all experiments and analyzed data. S-YL, Y-MC, S-JO, S-BY, and JL edited the manuscript. W-HC provided clinical expertise and samples. Y-HK and B-JK interpreted the experiments. S-YL, Y-MC, and B-JK wrote manuscript.

### Conflict of Interest Statement

The authors declare that the research was conducted in the absence of any commercial or financial relationships that could be construed as a potential conflict of interest. The reviewer KW and handling editor declared their shared affiliation.
