# Supplementary material for: rt269I Type of Hepatitis B Virus (HBV) Leads to HBV e Antigen Negative Infections and Liver Disease Progression via Mitochondrial Stress Mediated Type I Interferon Production in Chronic Patients With Genotype C Infections
Source: Front Immunol. 2019 Jul 24;10:1735. doi: 10.3389/fimmu.2019.01735 (PMC6677150; doi:10.3389/fimmu.2019.01735)
Supplement: Supplementary file 1 [file Table_1.DOCX]

**Supplemental Materials for**

**This file includes:**

Supplementary Table S1 and S2

Supplementary Figure S1 to S5

**SUPPLEMENTARY TABLES**

| **Pos**  **(1-344)** | **Stanford HBV reverse transcriptase database** | | | | | | | | | **131 patients** | **Polymorphism** |
| --- | --- | --- | --- | --- | --- | --- | --- | --- | --- | --- | --- |
|  | **A (n=506)** | **B (n=552)** | **C (n=734)** | **D (n=887)** | **E (n=311)** | **F (n=132)** | **G (n=22)** | **H (n=27)** | **I (n=52)** |  |  |
| **7** | **D**  A (17.8%)  V (12.5%)  T (10.6%) | **T**  A (19.0%)  S (1.4%) | **T**  A (4.4%)  I (0.8%) | **A**  T (4.3%)  V (2.2%)  D (1.4%) | **T**  S (0.7%) | **Y**  H (18.7%)  N (1.1%) | **T**  D (5.6%) | **Y** | **T**  N (52.1%)  S (4.2%)  Y (2.1%) | **T**  N (0.7%) | **T**  **Y**  **A**  **D** |
| **13** | **H**  Y (0.7%)  N (0.2%) | **R**  H (3.7%)  L (2.2%) | **N**  H (23.3%)  S (3.6%) | **H**  N (0.4%)  Y (0.4%) | **H**  Y (1.3%) | **H**  Y (22.0%) | **H** | **H** | **H**  Y (1.9%) | **N**  H (4.5%)  T (1.5%) | **H**  **N**  **R** |
| **55** | **R**  S (0.2%) | **R**  H (0.3%) | **H**  R (6.3%)  Q (6.2%) | **R**  K (0.1%) | **R** | **R**  Q (0.8%) | **R** | **R** | **R**  H (2.0%) | **H**  R (10.6%)  Q (5.3%)  K (1.5%) | **R**  **H** |
| **139** | **Q**  E (0.2%) | **N**  H (1.8%)  K (1.5%) | **H**  K (1.4%)  T (0.7%) | **N**  S (0.8%)  T (0.5%) | **N**  D (1.3%) | **N**  H (0.8%) | **Q** | **N** | **Q**  N (3.8%) | **H**  K (3.0%)  T (0.7%) | **N**  **Q**  **H** |
| **269** | **I**  L (2.5%) | **I**  L (2.0%) | **I**  L (43.0%) | **I**  L (2.6%) | **I** | **I**  L (5.7%) | **I** | **I** | **I** | **I**  L (57.2%) | **I**  **L** |
| **337** | **N**  D (2.1%)  H (0.5%) | **N**  T (9.2%)  H (2.5%) | **N**  H (19.5%)  T (0.9%) | **N**  T (3.5%)  H (1.3%) | **N**  T (2.7%)  D (2.3%) | **N**  H (8.0%) | **N** | **N**  T (3.7%) | **N**  H (6.4%) | **H**  N (11.4%) | **N**  **H** |

**Supplementary Table S1.** Definition of HBV mutation and polymorphism at reverse transcriptase based on database and cohort data.

**Supplementary Table S2.** PCR primers used for this study.

| **Primer** | **Forward** | **Reverse** |
| --- | --- | --- |
| POL-RT1 | CAG CCT ACT CCC ATC TCT CCA CCT CTA AG-3 | GCT CCA GAC CGG CTG CGA GC-3 |
| POL-RT2 | CCT CAG GCC ATG CAG TGG AA | GTA TGG ATC GGC AGA GGA GC |
| rt269I-C | GAA CAT ATT GTA CAA AAA ATC AAG CAA TGT TTT CGG AAA | TTT CCG AAA ACA TTG CTT GAT TTT TTG TAC AAT ATG TTC |
| pgRNA | GGT CCC CTA GAA GAA GAA CTC CCT | CAT TGA GAT TCC CGA GAT TGA GAT |
| cccDNA | CCG TCT GTG CCT TCT CAT | CAC AGC TTG GAG GCT TGA AC |
| mouse APOBEC3G | CCC CTG TTT CGA ATG TGC A | TGG TGT GTA GCC AGG AAC CTT |
| mouse IFN-α | TCT GAT GCA GCA GGT GGG | AGG GCT CTC CAG ACT TCT GCT CTG |
| mouse IFN-β | GCA CTG GGT GGA ATG AGA CT | AGT GGA GAG CAG TTG AGG ACA |
| mouse iNOS | GGC AGC CTG TGA GAC CTT TG | GCA TTG GAA GTG AAG CGT TTC |
| human APOBEC3G | ACA ACA GGT AGT AGG CGA CA | CCA ACA GTG CTG AAA TTC GTC ATA |
| human IFN-β | AGC AAG TTG TAG CTC ATG GAA | ACA ACA GGT AGT AGG CGA CA |
| human RIG-I | GGA CGT GGC AAA ACA AAT CAG | GCA ATG TCA ATG CCT TCA TCA |
| human ISG-15 | AGC TCC ATG TCG GTG TCA G | GAA GGT CAG CCA GAA CAG GT |
| human HO-1 | ACA TCT ATG TGG CCC TGG AG | TGT TGG GGA AGG TGA AGA AG |
| human iNOS | GTT CTC AAG GCA CAG GTC TC | GCA GGT CAC TTA TGT CAC TTA TC |
| mouse/human 18S rRNA | AGT CCC TGC CCT TTG TAC ACA | CGA TCC GAG GGC CTC ACT A |

**SUPPLEMENTARY FIGURES**

**
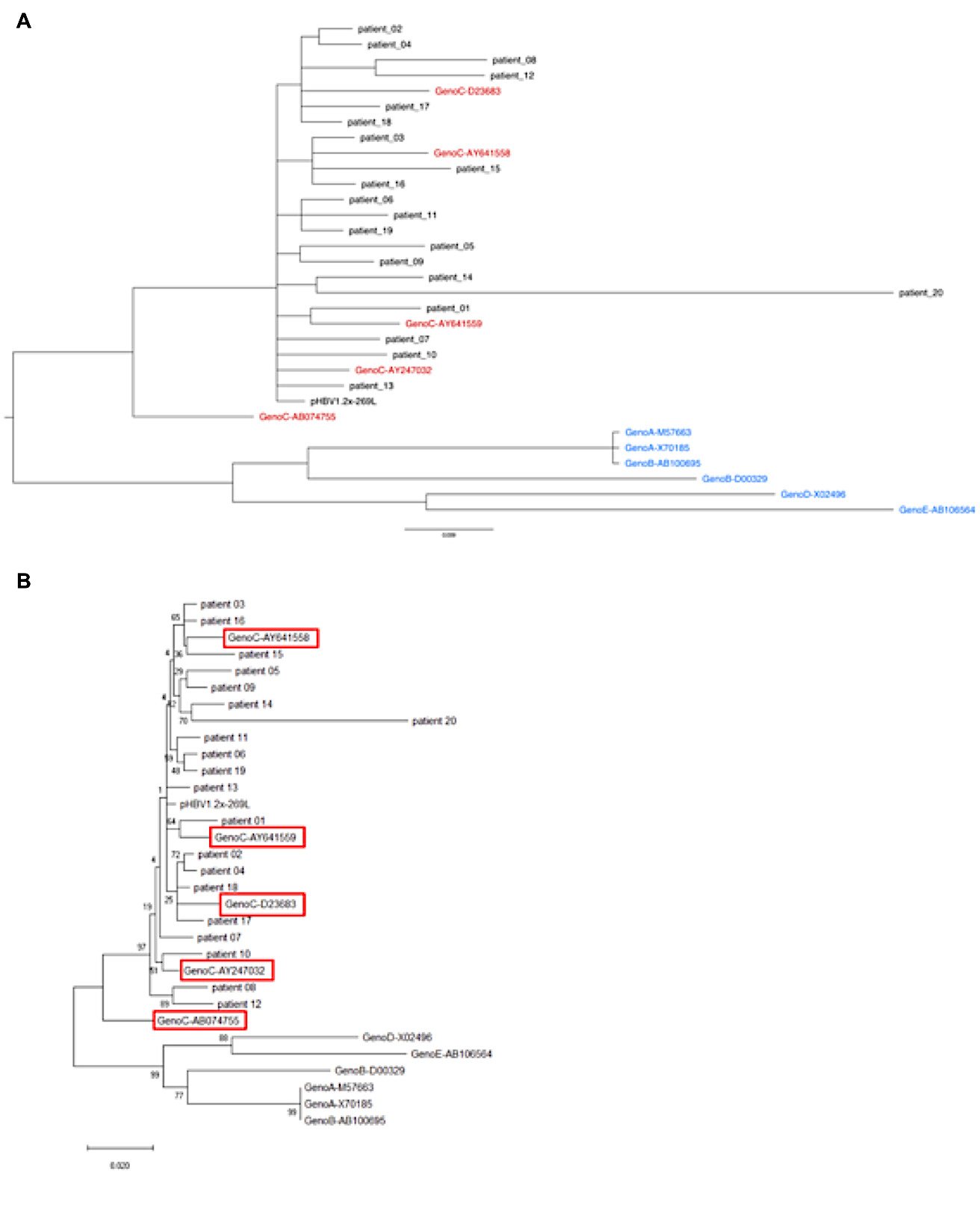
FIGURE S1.** Phylogenetic analyses of 1,032-bp polymerase RT sequences showed that the representative 20 patients used in cohort study belonged to genotype C. The RT sequences were aligned using MEGA7 and the phylogenetic trees were constructed using Bayesian (**A**) and Maximum likelihood (**B**) method. The bootstrap values were calculated from 1,000 replications, and burn in was set at 25% of samples. The bars indicate numbers of substitutions per nucleotide position.

**FIGURE S2** rt269I showed reduced features of viral replication in different cell lines. (**A**) Linear DNA was transfected to HepG2 cells and the levels of secreted HBsAg and HBeAg were analyzed using ELISA and cccDNA level were determined qPCR. (**B**) HBV DNA was transfected into Huh7 cells and replication factors were determined. HBsAg were measured by ELISA and extracellular viral DNA, cccDNA, and pgRNA levels were measured using ELISA and qPCR at 48 hours post-transfection. (**C**) Plasmid DNA with genotype C rt269L or rt269I was transfected in Huh-7.5 cells and HBsAg and extracellular viral DNA levels were measured using ELISA and qPCR at 48 hours after transfection, respectively. These data were normalized with β-Galactosidase assay. Data represent mean±S.D. One- and two-way ANOVA were used. ^*^*p*<0.05, ^**^*p*<0.01, ^***^*p*<0.001.

**FIGURE S3** rt269I enhanced IFN-I production in Huh7 cells. HBV DNA and pSV-β-Galactosidase was transfected into Huh7 cells and the expression levels of IFN-I-related genes were assessed. The mRNA level of IFN- β, RIG-I, and ISG-15 were determined using qRT-PCR at 12 hours after transfection. β-Galactosidase enzyme assay was assessed to normalization. Data represents mean ± S.D. of three independent experiments. One-way ANOVA were used. ^*^*p*<0.05, ^**^*p*<0.01, ^***^*p*<0.001.

**FIGURE S4.** IFN-I was enhanced by rt269I. (**A**-**D**) The culture supernatant was collected at 24 hours post-transfection and used to measure IFN-I using a luciferase assay with HEK293 cells with the construct of the luciferase reporter gene under the control of IRSE promoter. 1.2x-mock, rt269L, and rt269I plasmid DNA (genotype C) were transfected into the HepG2 (**A**), Huh 7 (**B**) and Huh 7.5 cells (**C**). (**D**) pHY92-rt269L (genotype A), 1.2x-rt269L (genotype C), and 1.2x-rt269I (genotype C) plasmid DNA were transfected into the HepG2 cells and the IFN-I levels were compared among the groups. (**E)** HBV DNA were transfected into mouse hepatoma cell Hepa1c1c-7, and mouse IFN- β in the supernatants were measured by ELISA. These data were normalized with β-Galactosidase assay. Data represent mean±S.D. One- and two-way ANOVA were used. ^*^*p*<0.05, ^**^*p*<0.01, ^***^*p*<0.001


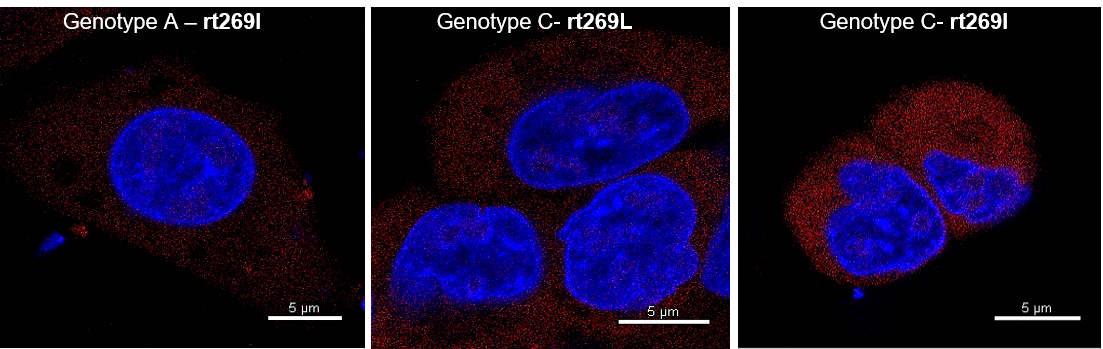


**FIGURE S5.** rt269I induced mitochondrial reactive oxygen species production. The secreted mtROS levels were analyzed using MitoSox via an immunofluorescence assay at 24 hours after transfection. The HepG2 cells were transfected with pHY92-rt269L (genotype A), 1.2x-rt269L (genotype C), and 1.2x-rt269I (genotype C) plasmid DNA.
